# Metabolism of Non-Enzymatically Derived Oxysterols: Clues from sterol metabolic disorders^☆^^☆☆^

**DOI:** 10.1016/j.freeradbiomed.2019.04.020

**Published:** 2019-11-20

**Authors:** William J. Griffiths, Eylan Yutuc, Jonas Abdel-Khalik, Peter J. Crick, Thomas Hearn, Alison Dickson, Brian W. Bigger, Teresa Hoi-Yee Wu, Anu Goenka, Arunabha Ghosh, Simon A. Jones, Douglas F. Covey, Daniel S. Ory, Yuqin Wang

**Affiliations:** a Institute of Life Science, Swansea University Medical School, Singleton Park, Swansea, SA2 8PP, UK; b Stem Cell & Neurotherapies, Division of Cell Matrix Biology and Regenerative Medicine, Stopford Building, Oxford Road, University of Manchester, Manchester, M13 9PT, UK; c Manchester Centre for Genomic Medicine, 6th Floor, St Mary's Hospital, Central Manchester Foundation Trust, University of Manchester, Oxford Road, Manchester, M13 9WL, UK; d Department of Developmental Biology, Washington University School of Medicine, St Louis, MO, 63110, USA; e Diabetic Cardiovascular Disease Center, Washington University School of Medicine, St. Louis, MO, 63110, USA

**Keywords:** Niemann-Pick disease, Cholesterol/metabolism, Bile acid and salts/biosynthesis, Inborn errors of metabolism, Mass spectrometry, Lipidomics, Free radical

## Abstract

Cholestane-3β,5α,6β-triol (3β,5α,6β-triol) is formed from cholestan-5,6-epoxide (5,6-EC) in a reaction catalysed by cholesterol epoxide hydrolase, following formation of 5,6-EC through free radical oxidation of cholesterol. 7-Oxocholesterol (7-OC) and 7β-hydroxycholesterol (7β-HC) can also be formed by free radical oxidation of cholesterol. Here we investigate how 3β,5α,6β-triol, 7-OC and 7β-HC are metabolised to bile acids. We show, by monitoring oxysterol metabolites in plasma samples rich in 3β,5α,6β-triol, 7-OC and 7β-HC, that these three oxysterols fall into novel branches of the acidic pathway of bile acid biosynthesis becoming (25R)26-hydroxylated then carboxylated, 24-hydroxylated and side-chain shortened to give the final products 3β,5α,6β-trihydroxycholanoic, 3β-hydroxy-7-oxochol-5-enoic and 3β,7β-dihydroxychol-5-enoic acids, respectively. The intermediates in these pathways may be causative of some phenotypical features of, and/or have diagnostic value for, the lysosomal storage diseases, Niemann Pick types C and B and lysosomal acid lipase deficiency. Free radical derived oxysterols are metabolised in human to unusual bile acids via novel branches of the acidic pathway, intermediates in these pathways are observed in plasma.

## Highlights

• Free radical derived oxysterols metabolised to unusual bile acids.
• Metabolism proceeds via novel branches of the acidic pathway.
• Bile acids with:- 3β,5α,6β-triol; 3β,7β-diol-5-ene or 3β-ol-5-en-7-one in A/B-ring.
• Unusual bile acids found in plasma from patients with NPC, NPB and LALD.
• Intermediates in the biosynthesis of unusual bile acids found in plasma.

## Graphical abstract

**Figure:**
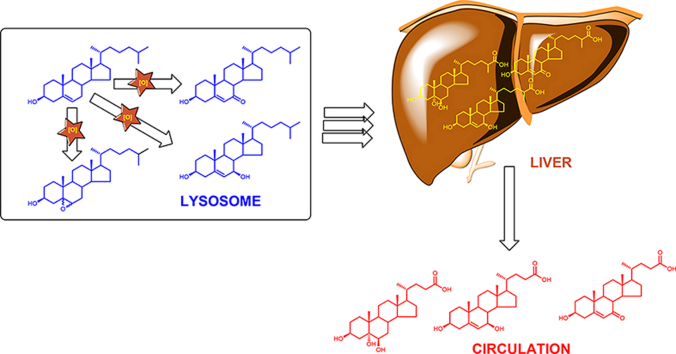

## Abbreviations

ACOT: acyl-CoA thioesterases
ACOX2: acyl-coenzyme A oxidase 2
AMACR: alpha-methylacyl-CoA-racemase
BACS: bile acyl CoA-synthetase
ChEH: cholesterol epoxide hydrolase
ChOx: cholesterol oxidase
CYP: cytochrome P450
DBP: D bifunctional protein
DDA: dendrogenin A
Hh: Hedgehog
HSD: hydroxysteroid dehydrogenase
GlcNAc: N-acetylglucosamine
GP: Girard P reagent
LALD: lysosomal acid lipase deficiency
LC-MS: liquid chromatography mass spectrometry
MRM: multiple reaction monitoring
MS^n^: mass spectrometry with multistage fragmentation
NPA: Niemann-Pick disease type A
NPB: Niemann-Pick disease type B
NPC: Niemann-Pick disease type C
RICs: reconstructed ion chromatograms
SLOS: Smith-Lemli-Opitz syndrome
Smo: Smoothened
SPCx: sterol carrier protein x
3β-HCA: 3β-hydroxycholest-5-en-(25R)26-oic acid
3βH,7O-CA: 3β-hydroxy-7-oxocholest-5-en-(25R)26-oic acid
3βH,7O-Δ^5^-BA: 3β-hydroxy-7-oxochol-5-enoic acid
3β,5α-diHC-6O: 3β,5α-dihydroxycholestan-6-one
3β,5α,6β-triHBA: 3β,5α,6β-trihydroxycholanoic acid
3β,5α,6β-triHCa: 3β,5α,6β-trihydroxycholestan-(25R)26-oic acid
3β,5α,6β-triol: cholestane-3β,5α,6β-triol
3β,5α,6β,24-tetraHCa: 3β,5α,6β,24-tetrahydroxycholestan-26-oic acid
3β,5α,6β,26-tetrol: cholestane-3β,5α,6β,(25R)26-tetrol
3β,7α-diHCA: 3β,7α-dihydroxycholest-5-en-(25R)26-oic acid
3β,7β-diHCA: 3β,7β-dihydroxycholest-5-en-(25R)26-oic acid
3β,7β-diH-Δ^5^-BA: 3β,7β-dihydroxychol-5-enoic acid
3β,7β,24-triHCA: 3β,7β,24-trihydroxycholest-5-en-26-oic acid
3β,24-diH,7O-CA: 3β,24-dihydroxy-7-oxocholest-5-en-26-oic acid
5α,6-EC: 5α,6-epoxycholesterol
5β,6-EC: 5β,6-epoxycholesterol
7-DHC: 7-dehydrocholesterol
7-OC: 7-oxocholesterol
7α-HCO: 7α-hydroxycholest-4-en-3-one
7α-HC: 7α-hydroxycholesterol
7αH,3O-CA: 7α-hydroxy-3-oxocholest-4-en-(25R)26-oic acid
7β-HC: 7β-hydroxycholesterol
7β,26-diHC: 7β,26-dihydroxycholesterol
24S-HC: 24S-hydroxycholesterol
25-HC: 25-hydroxycholesterol
25H,7O-C: 25-hydroxy-7-oxocholesterol
26-HC: (25R)26-hydroxycholesterol
26H,7O-C: 26-hydroxy-7-oxocholesterol.

## 1 Introduction

**Fig. 1:**
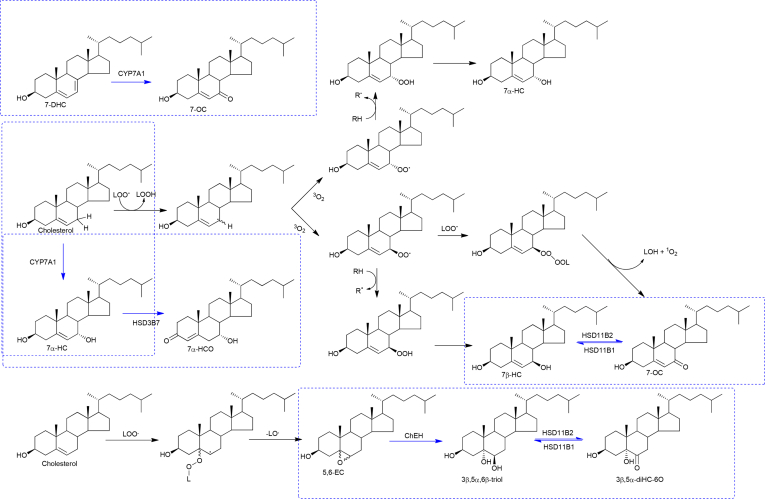
Free radical formation of 7α-HC, 7β-HC, 7-OC and 5,6-EC [34]. The reactions shown in blue boxes and by blue arrows are enzyme catalysed. CYP, cytochrome P450; HSD, hydroxysteroid dehydrogenase; ChEH, cholesterol epoxide hydrolase; LOO^**.**^, lipidperoxy radical; LO^**.**^, lipidalkoxy radical; LOH, lipidhydroxide; LOOH lipidhydroperoxide; RH, hydrogen donor.

The oxysterols 7-oxocholesterol (7-OC, for systematic names see Supplemental Table S1), 7β-hydroxycholesterol (7β-HC), 5α,6-epoxycholesterol (5α,6-EC), 5β,6-epoxycholesterol (5β,6-EC) and cholestane-3β,5α,6β-triol (3β,5α,6β-triol) have for many years been thought of as autoxidation artefacts formed *ex vivo* from cholesterol during sample handling [[1], [2], [3]]. This view is now changing with the realisation that (i) 7-OC can be formed enzymatically from 7-dehydrocholesterol (7-DHC) [4], (ii) 7β-HC and 7-OC can be interconverted in enzyme catalysed reactions [[5], [6], [7], [8], [9]], while (iii) the 5α,6-EC adduct dendrogenin A (DDA) is present in tissue [10], (iv) 5,6-EC is hydrolysed by the enzyme cholesterol epoxide hydrolase (ChEH) to 3β,5α,6β-triol which itself is oxidised by hydroxysteroid dehydrogenase (HSD) 11B2 to 3β,5α-dihydroxycholestan-6-one (3β,5α-diHC-6O, also called 6-oxocholestan-3β,5α-diol) found at elevated levels in breast cancer tissue [11], and (v) high levels of 7-OC and 3β,5α,6β-triol are characteristic of the lysosomal storage diseases Niemann-Pick types A, B, C1 and C2 (NPA, NPB, NPC) and lysosomal acid lipase deficiency (LALD) [[12], [13], [14], [15], [16], [17], [18]]. In these inborn errors of metabolism, characterised by the accumulations of multiple lipid species including the build-up of lysosomal cholesterol, it is likely that 7-OC and 5,6-EC, the precursor of 3β,5α,6β-triol, are formed via free radical reactions (Fig. 1). Importantly, 7-OC, 7β-HC, 5α,6-EC and 3β,5α,6β-triol are precursors of biologically active molecules, including 25-hydroxy-7-oxocholesterol (25H,7O-C) and 26-hydroxy-7-oxocholesterol (26H,7O-C) which are ligands to the Hedgehog (Hh) pathway oncoprotein Smoothened (Smo) [19]; 7β,26-dihydroxycholesterol (7β,26-diHC), a ligand to the nuclear receptor RORγt [20]; DDA a tumour suppressor [21] and both DDA and dendrogenin B have potent proliferative effects in neural stem cells [10]; and the oncometabolite and glucocorticoid receptor ligand 3β,5α-diHC-6O [11]. Accepting that the 5,6-epoxides and 7-oxidised sterols are formed *in vivo*, interest now shifts to investigating how they are metabolised. With respect to 7-OC, we have recently reported that in patients with the recessive congenital disorder Smith-Lemli-Opitz syndrome (SLOS) 7-OC can be converted to 7β-hydroxy bile acids and ultimately conjugated with *N-*acetylglucosamine (GlcNAc) [[22], [23], [24], [25]]. A similar pathway may also be operative in patients with NPC disease where 7-OC is also elevated and the same GlcNAc conjugated bile acids have been identified [26]. Patients with NPC, NPB or NPA show elevated 3β,5α,6β-triol in plasma, presumably derived from free radical oxidation of cholesterol to 5,6-EC followed by hydrolysis to the triol by ChEH (Fig. 1) [27]. Clayton and colleagues and Ory and colleagues have now both identified 3β,5α,6β-trihydroxycholanoic acid (3β,5α,6β-triHBA) as the bile acid product of 3β,5α,6β-triol metabolism [28,29]. An alternative route for metabolism of 3β,5α,6β-triol is oxidation to the oncometabolite 3β,5α-diHC-6O [11].

Although the end products of 7-OC and 5,6-EC/3β,5α,6β-triol metabolism have been defined, the *in vivo* biochemical pathways generating these products have yet to be fully elucidated. To study further the metabolism of 7-OC and 5,6-EC/3β,5α,6β-triol we took advantage of plasma samples from patients where levels of these substrates are particularly high. We find that 7-OC, 7β-HC and 3β,5α,6β-triol fall into new branches of the acidic pathway of bile acid biosynthesis and in patient plasma where the concentration of these metabolites is high, almost all the necessary intermediates to bile acids are observed. Build-up of these intermediates may be responsible for some of the clinical features of diseases where free radical oxidation of cholesterol is prevalent and their measurement may have diagnostic value.

## 2 Materials and methods

### 2.1 Materials

Oxysterol standards were from Avanti Polar Lipids Inc (Alabaster, Al, USA), 3β,5α,6β-triHBA was prepared as in Ref. [29], other bile acid standards were kindly donated by Dr Jan Sjövall of Karolinska Institute, Stockholm, or as detailed in Griffiths et al. [30]. Materials for liquid chromatography – mass spectrometry (LC-MS) analysis were as in Refs. [31,32].

### 2.2 Patient samples

All participants or their parents/guardians provided informed consent and the study was performed with institutional review board approval (REC08/H1010/63) and adhered to the principles of the Declaration of Helsinki.

### 2.3 LC-MS methods

**Fig. 2:**
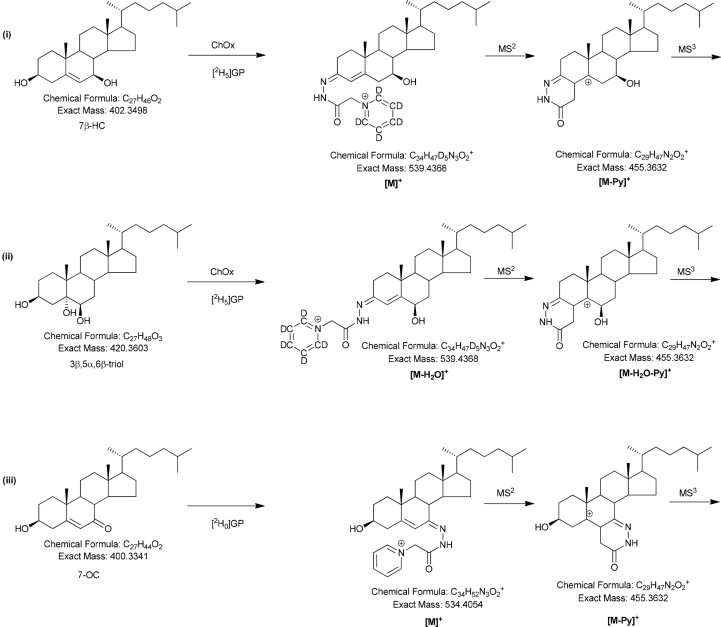
Derivatisation of (i) 3β-hydroxy-5-ene, and (ii) 3β,5α,6β-triol, containing sterols via oxidation with bacterial cholesterol oxidase (ChOx) and reaction with [^2^H_5_]GP, and of (iii) 7-oxo-sterols by reaction with [^2^H_0_]GP in the absence of cholesterol oxidase. The reactions are illustrated for 7β-HC, 3β,5α,6β-triol and 7-OC, respectively.

The LC-MS methods have been described in detail elsewhere [30,31]. In brief, a charge-tagging method was adopted [31,32], where sterols, including oxysterols and bile acids, with a 3β-hydroxy group were oxidised with bacterial cholesterol oxidase (ChOx) to 3-oxo analogues and derivatised with [^2^H_5_]-labelled Girard P (GP) reagent (Fig. 2), then analysed by LC-MS at high mass-resolution (120,000 at *m/z* 400, full-width at half-maximum height definition) with parallel multistage fragmentation (MS^n^). Sterols with a natural oxo group were derivatised with [^2^H_0_]GP reagent in the absence of cholesterol oxidase and analysed together with the [^2^H_5_]GP-derivatised sterols in a single LC-MS(MS^n^) run. Quantification was by the isotope dilution method.

## 3 Results

### *3.1* *GP derivatisation of 3*β*-hydroxy-5-enesterols, 3*β*,5α,6*β*-trihydroxysterols and 7-oxosterols*

Cholesterol oxidase from *Streptomyces* sp converts sterols with a 3β-hydroxy-5-ene function to analogues with a 3-oxo-4-ene structure, which are substrates for derivatisation with [^2^H_5_]GP (Fig. 2). The derivatisation products give [M]^+^ ions in LC-MS analysis. Upon oxidation/derivatisation of 3β,5α,6β-trihydroxysterols the 5α-hydroxy group is labile with the result that these oxysterols becomes converted to [^2^H_5_]GP-derivatives of a 6β-hydroxy-3-oxo-4-ene structure, i.e. the [M − H_2_O]^+^ ion of the oxidised/derivatised triol [33]. 7-Oxosterols do not require cholesterol oxidase treatment prior to [^2^H_0_]GP derivatisation and are hence evident as [M]^+^ ions in preparations in the absence of enzyme (Fig. 2). GP-derivatised sterols containing a 3β,5α,6β-triol function give characteristic MS^3^ spectra this allows their presumptive identification by LC-MS(MS^n^) in the absence of authentic standards. This is also true for 7-oxo- and 3β,7β-dihydroxysterols [30].

### 3.2 Metabolism of 5,6-EC

**Fig. 3:**
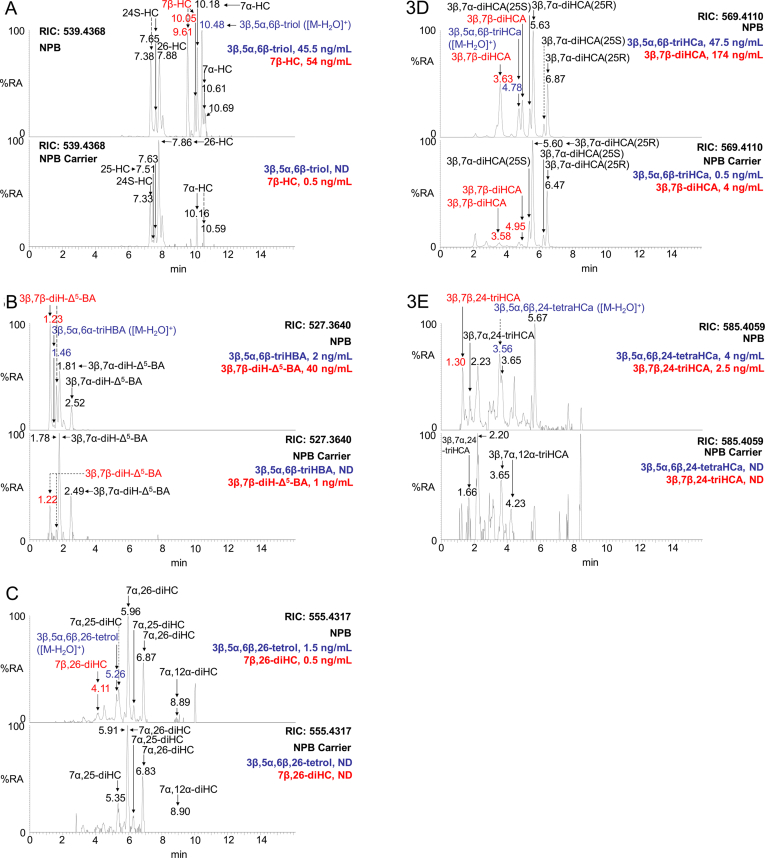
LC-MS reconstructed ion chromatograms (RICs) of oxidised/[^2^H_5_]GP derivatised oxysterols and bile acids in plasma from an NPB patient and NPB carrier. RICs for (A) *m/z* 539.4368 corresponding to [M]^+^ ions of hydroxycholesterols (HC) and [M − H_2_O]^+^ ions of 3β,5α,6β-triol, (B) *m/z* 527.3640 corresponding to [M]^+^ ions of dihydroxychol-5-enoic acids (diH-Δ^5^-BA) and [M − H_2_O]^+^ ions of 3β,5α,6β-triHBA, (C) *m/z* 555.4317 corresponding to [M]^+^ ions of dihydroxycholesterols (diHC) and [M − H_2_O]^+^ ions of 3β,5α,6β,26-tetrol, (D) *m/z* 569.4110 corresponding to [M]^+^ ions of dihydroxycholest-5-en-26-oic acids (diHCA) and [M − H_2_O]^+^ ions of 3β,5α,6β-triHCa, (E) *m/z* 585.4059 corresponding to trihydroxycholest-5-en-26-oic acids (triHCA) and [M − H_2_O]^+^ ions of 3β,5α,6β,24-tetraHCa. The upper panels show RICs of NPB patient and lower panels NPB carrier plasma. Oxysterols with a the 3β,5α,6β-triol function are labelled in blue, those with a 3β,7β-dihydroxy function in red. Concentrations are given in the righthand corner of chromatograms. GP derivatised oxysterols give *syn* and *anti* conformers, which may or may not be chromatographically separated. For quantification of 3β,5α,6β,26-tetrol and 3β,5α,6β,24-tetraHCa extended chromatographic gradients were exploited as described in Griffiths et al. [30].

Both 5α,6-EC and 5β,6-EC are converted *in vivo* by ChEH to 3β,5α,6β-triol [34,35] (Fig. 1) and both Mazzacuva et al. [28] and Jiang et al. [29] have found 3β,5α,6β-triHBA to be a bile acid produced by NPC patients in whom the 3β,5α,6β-triol is abundant. We thus sought to confirm these finding by analysis of NPC patient plasma (n = 3) and to identify the intermediates in the biochemical pathway from 3β,5α,6β-triol to 3β,5α,6β-triHBA. 3β,5α,6β-triol is also reported to be abundant in plasma samples from NPB and LALD patients [[15], [16], [17], [18]], so we similarly analysed plasma samples from patients with these disorders (NPB, n = 3; LALD, n = 2). We also analysed plasma samples from carriers of these disorders (see Supplemental Table S1). To minimise problems of *ex vivo* oxidation of cholesterol producing 5,6-EC artefacts, cholesterol was removed from samples by reversed-phase solid phase extraction as a first step in sample preparation, consequently 5,6-EC and 3β,5α,6β-triol, the latter of which may be formed by *ex vivo* acid catalysed hydrolysis of 5,6-EC, are minimised in plasma samples from healthy donors, including disease carriers (5,6-EC <0.5 ng/mL; 3β,5α,6β-triol <3.5 ng/mL), see also Supplemental Table S1 which embraces data from the standard reference material NIST SRM 1950, prepared from plasma samples from 100 individuals between 40 and 50 years of age, whose ethnicity was representative of the US population and which included an equal number of men and women [36]. However, upon analysis of patient plasma for 3β,5α,6β-triol, elevated levels were found in NPC (range 31–53 ng/mL) and NPB (23.5–45.5 ng/mL) samples (Supplemental Table S1, Fig. 3A) confirming the results of earlier studies [13,[15], [16], [17], [18]], although the triol was only increased in one of the LALD samples (3.5–7.5 ng/mL). The precursor of 3β,5α,6β-triol, 5,6-EC, was similarly elevated in NPC (1–2.5 ng/mL) plasma, although in only one of the NPB (0.5–2 ng/mL) and LALD (0.5–1 ng/mL) plasma samples. The product of 3β,5α,6β-triol metabolism, 3β,5α,6β-triHBA, was found in NPC (1–3.5 ng/mL), NPB (1–2 ng/mL) and one of the two LALD (0–1.5 ng/mL) plasma samples (Fig. 3B), but was absent from controls and carriers (≤0.1 ng/mL). It is likely that 3β,5α,6β-triol is converted to 3β,5α,6β-triHBA in a series of enzyme catalysed reactions, like those operative in the acidic pathway of bile acid biosynthesis [37].

#### 3.2.1 Acidic pathway

**Fig. 4:**
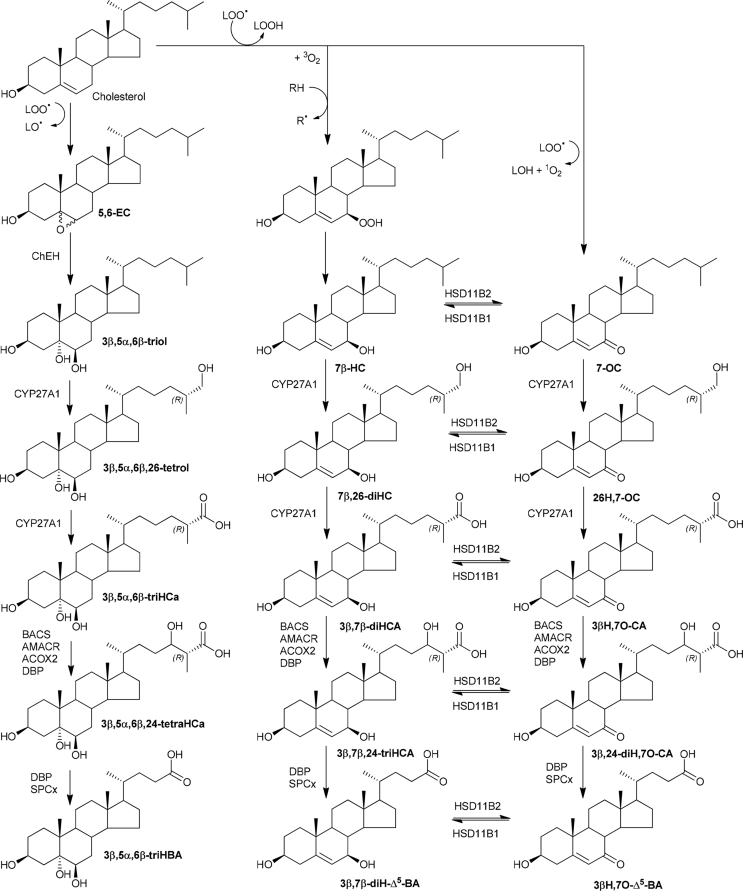
Formation and metabolism of 5,6-EC, 7β-HC and 7-OC. The enzymes catalysing the reactions are cholesterol epoxide hydrolase (ChEH), hydroxysteroid dehydrogenase 11B1 (HSD11B1), HSD11B2, cytochrome P450 27A1 (CYP27A1), bile acyl CoA-synthetase (BACS), alpha-methylacyl-CoA-racemase (AMACR), acyl-coenzyme A oxidase 2 (ACOX2), D bifunctional protein (DBP) and sterol carrier protein x (SPCx). For simplicity, the individual reactions catalysed by BACS, AMACR, ACOX2, DBP and SPCx are omitted and carboxylic acids rather than CoA-thioesters are shown. The peroxisomal enzyme bile acid CoA thioesterase (ACOT) will hydrolyse the CoA-thioesters to free acids [38,39].

If 3β,5α,6β-triol falls into a branch of the acidic pathway of bile acid biosynthesis the next metabolite, generated by the enzyme cytochrome P450 (CYP) 27A1, should be cholestane-3β,5α,6β,(25R)26-tetrol (3β,5α,6β,26-tetrol, Fig. 4). Although we do not have an authentic standard of the tetrol, it was presumptively identified in plasma samples from patients with NPC (0.5–1.5 ng/mL) and NPB (1–1.5 ng/mL) based on LC retention time, exact mass and MS^3^ spectrum, but was absent from control and carrier plasma (Fig. 3C, see Figure 6G in Ref. [30] for MS^3^ spectrum). Further, metabolism catalysed by CYP27A1 should lead to 3β,5α,6β-trihydroxycholestan-(25R)26-oic acid (3β,5α,6β-triHCa). Evidence for the presence of 3β,5α,6β-triHCa in plasma samples from patients with NPC (6–16 ng/mL), NPB (31.5–47.5 ng/mL) and LALD (2–21 ng/mL) was again provided by LC retention time, exact mass and MS^3^ spectra (see Figure 6D in Ref. [30] for MS^3^ spectrum). 3β,5α,6β-triHCa was present at low levels in control and carrier plasma (≤0.5 ng/mL, Fig. 3D). It is noteworthy that in the separate patient groups the concentrations of this acid appears to reflect those of the triol. However, the highest concentration of 3β,5α,6β-triHCa in NPC plasma is only about half that of the lowest concentration in NPB plasma. Interestingly, the concentration of 3β-hydroxycholest-5-en(25R)26-oic (3β-HCA), 3β,7α-dihydroxycholest-5-en-(25R)26-oic (3β,7α-diHCA) and 7α-hydroxy-3-oxocholest-4-en-(25R)26-oic (7αH,3O-CA) acids, three cholestenoic acids of the conventional acidic pathway, are also higher in the NPB plasma samples (3β-HCA, 300–648 ng/mL; 3β,7α-diHCA, 132–336 ng/mL; 7αH,3O-CA, 120.5–316 ng/mL) than in NPC (3β-HCA, 54.5–111 ng/mL; 3β,7α-diHCA, 13.5–42 ng/mL; 7αH,3O-CA, 29.5–76 ng/mL) or controls and carriers (3β-HCA, 53–143.5 ng/mL; 3β,7α-diHCA 10.5–35.5 ng/mL; 7αH,3O-CA, 43.5–69 ng/mL). Importantly, the initial metabolite of the conventional acidic pathway, (25R)26-hydroxycholesterol (26-HC), formed by CYP27A1 metabolism of cholesterol, was higher in NPB plasma samples (40–68 ng/mL) samples than NPC (12–16 ng/mL), LALD (7.5–11.5 ng/mL) or controls and carriers (16–28 ng/mL). These trends were not observed for initial members of the neutral pathway of bile acid biosynthesis i.e. 7α-hydroxycholesterol (7α-HC, NPB, 10–12.5 ng/mL; NPC, 12–35 ng/mL; LALD, 2.5–3 ng/mL; control/carrier, 1.5–13.5 ng/mL), the 24-hydroxylase pathway i.e. 24S-hydroxycholesterol (24S-HC, NPB, 9–34.5 ng/mL; NPC, 11.5–15.5 ng/mL; LALD, 28.5–34.5 ng/mL; control/carriers, 7–13.5 ng/mL) or the 25-hydroxylase pathway i.e. 25-hydroxycholesterol (25-HC, NPB, 1.5–2 ng/mL; NPC, 1–1.5 ng/mL; LALD <1 ng/mL, control/carriers, 0.5–1 ng/mL).

### 3.3 Shortening C_27_ to C_24_ bile acids

Side-chain shortening of 3β,5α,6β-triHCa is likely to proceed in the peroxisome. This would involve formation of a CoA-thioester via the enzyme bile acyl CoA-synthetase (BACS), its epimerisation at C-25 by alpha-methylacyl-CoA-racemase (AMACR), dehydrogenation of the side-chain between C-24 and C-25 by acyl-coenzyme A oxidase 2 (ACOX2), then hydration of the resultant double bond by peroxisomal multifunctional enzyme type 2, also known as D bifunctional protein (DBP), to give 3β,5α,6β,24-tetrahydroxycholestan-26-oic acid (3β,5α,6β,24-tetraHCa) as the CoA thioester (in Fig. 4, for simplicity the individual steps between 3β,5α,6β-triHCa and 3β,5α,6β,24-tetraHCa have been omitted and the free acids are shown rather than the CoA thioesters). Using our oxidation/derivatisation method the free acids are preferentially observed and, in the absence of an authentic standard, 3β,5α,6β,24-tetraHCa was presumptively identified by LC retention time, exact mass and MS^3^ spectrum in plasma samples from NPC (0.5–2.5 ng/mL), NPB (2.5–4 ng/mL) and LALD (0.5–4.5 ng/mL, see Supplemental Figure S5Q in Ref. [30]). There was no evidence for 3β,5α,6β,24-tetraHCa in control or carrier plasma (Fig. 3E). As with 3β,5α,6β-triHCa, the concentration of 3β,5α,6β,24-tetraHCa reflects that of the triol within separate patient groups, but not between groups where concentrations of the acids are higher in NPB plasma. The ultimate C_24_ bile acid CoA thioester should be formed by beta-oxidation through sterol carrier protein x (SPCx) after dehydrogenation by DBP (HSD17B4). As indicated above, free bile acids are preferentially observed with the current method and 3β,5α,6β-triHBA was detected in NPC (1–3.5 ng/mL), NPB (1–2 ng/mL) and one of the two LALD (0–1.5 ng/mL) patient samples, but not controls or carriers (≤0.1 ng/mL) (Fig. 3B). A peroxisomal CoA thioesterase can catalyse the hydrolysis of bile acid CoA thioesters into free bile acids [38,39].

### *3.4* *Metabolism of 7-OC and 7*β*-HC*

The metabolism of 7-OC has been of interest to many investigators. Lyons and Brown reported that it could be metabolised to 26H,7O-C by CYP27A1 in HepG2 cells, while Heo et al. showed that 26H,7O-C and the down-stream CYP27A1 metabolite 3β-hydroxy-7-oxocholest-5-en-(25R)26-oic acid (3βH,7O-CA) could be formed by retinal pigment epithelial cells [40,41]. An alternative route for metabolism of 7-OC is reduction to 7β-HC by HSD11B1 as shown by Hult et al., Larsson et al., Mitic et al. and Schweizer et al. [[5], [6], [7], [8]]. 7-OC itself can be formed via radical reactions from cholesterol or enzymatically from 7-DHC by CYP7A1 or from 7β-HC by HSD11B2 oxidation [4,9,34] (Fig. 1). We have reported a metabolic pathway from 7-OC to 7-oxo- and 7β-hydroxy-Δ^5^-bile acids in SLOS patients where the 7-DHC concentrations are high in plasma and tissue [[22], [23], [24], [25]]. We next sought to investigate if this pathway is active in other diseases where levels of 7-OC are elevated.

**Fig. 5:**
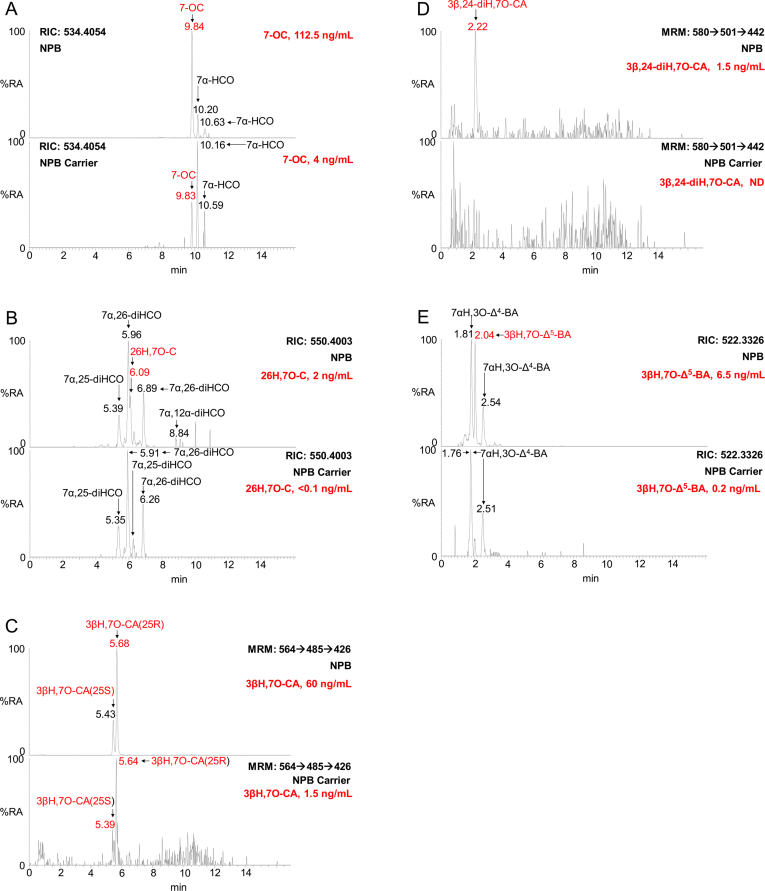
LC-MS RICs or MRM chromatograms of [^2^H_0_]GP derivatised oxysterols and bile acids from NPB patient and NPB carrier plasma. RICs of (A) *m/z* 534.4054 corresponding to [M]^+^ ions of 7α-hydroxycholest-4-en-3-one (7α-HCO) and 7-OC, (B) *m/z* 550.4003 corresponding to [M]^+^ ions of dihydroxycholest-4-en-3-ones (diHCO) and 26H,7O-C, and (E) *m/z* 522.3326 corresponding to [M]^+^ ions of hydroxy-3-oxochol-4-enoic acids (H,3O-Δ^4^-BA) and 3βH,7O-Δ^5^-BA. MRM chromatograms of (C) *m/z* 564 → 485→426 specific for 3βH,7O-CA, and (D) *m/z* 580 → 501→442 specific for 3β,24-diH,7O-CA. The upper panels show chromatograms of NPB patient and lower panels NPB carrier plasma. Oxysterols with a the 7-oxo function are labelled in red. Concentrations are given in the right-hand corner of chromatograms. GP derivatised oxysterols give *syn* and *anti* conformers, which may or may not be chromatographically separated. For quantification of 26H,7O-C an extended chromatographic gradient was exploited as described in Griffiths et al. [30].

Like 3β,5α,6β-triol, 7-OC is elevated in plasma of patients with lysosomal storage disorders. Patients with NPC and NPB show elevated levels of both 7-OC (NPC, 72.5–135.5 ng/mL; NPB 65.5–112.5 ng/mL, Fig. 5A) and 7β-HC (NPC, 29.5–69 ng/mL; NPB, 19–54 ng/mL, Fig. 3A) in plasma compared to controls and carrier (7-OC, <8.5 ng/mL; 7β-HC, <2 ng/mL). In plasma from LALD patients 7-OC (9.5–20 ng/mL) was just elevated but not 7β-HC (1.5–3.5 ng/mL). The ratio of 7-OC to 7β-HC was about 2.5 for the NPC (2.2 ± 0.3, mean ± standard deviation) and NPB (2.7 ± 0.7) patients, but much more variable for the carriers (12.9 ± 5.7) and LALD (6.10) patients. Interestingly, the ratio of 7-OC to 3β,5α,6β-triol was also about 2.5 for the NPC (2.2 ± 0.4), NPB (2.5 ± 0.2) and LALD (2.7) patients, but more variable for the carriers (3.3 ± 1.6). 7-OC and 7β-HC are interconverted by HSD11B1 and HSD11B2 and both may fall into branches of the acidic pathway of bile acid metabolism (Fig. 4), giving the bile acids 3β,7β-dihydroxychol-5-enoic (3β,7β-diH-Δ^5^-BA) and 3β-hydroxy-7-oxochol-5-enoic (3βH,7O-Δ^5^-BA) acid as metabolic products (see Supplemental Table S1). In fact, we observe almost all the necessary pathway intermediates in NPC, NPB and LALD patient plasma in contrast to controls and carriers where intermediates are absent or minor.

#### *3.4.1* *Acidic pathway:- 7*β*-HC*

Considering the metabolites of 7β-HC first, authentic standards are available for 7β-HC, 7β,26-diHC, 3β,7β-dihydroxycholest-5-en-(25R)26-oic acid (3β,7β-diHCA) and 3β,7β-diH-Δ^5^-BA and each are chromatographically resolved from their 7α-epimers [42]. 7β,26-diHC was absent from control and carrier plasma but was present in two of the three NPC (0–1.5 ng/mL) and NPB (0–0.5 ng/mL) and one of the two LALD (0–0.2 ng/mL) plasma samples (Fig. 3C). The further CYP27A1 metabolite, 3β,7β-diHCA, is a normal constituent of plasma from healthy individuals (control and carriers, 3–5 ng/mL), but is greatly elevated in NPC (40.5–229 ng/mL) and NPB (97–174 ng/mL) plasma and to a lesser extent in LALD (11–52.5 ng/mL) plasma (Fig. 3D). We do not have an authentic standard for 3β,7β,24-trihydroxycholest-5-en-26-oic acid (3β,7β,24-triHCA), however, the 7α-epimer (3β,7α,24-triHCA) is commercially available, this allows a presumptive, rather than definitive, identification of 3β,7β,24-triHCA based on exact mass, MS^3^ spectrum and retention time. The 7β-epimer is essentially absent from control and carrier plasma (<0.1 ng/mL), but present in two of the three NPC (0–1.5 ng/mL), all three of the NPB (2–4.5 ng/mL) and both of the LALD (0.5–2.5 ng/mL) plasma samples (Fig. 3E). The ultimate metabolite of the pathway, 3β,7β-diH-Δ^5^-BA was present in control and carrier plasma (0.5–2 ng/mL), but at higher levels in NPC (19–50 ng/mL), NPB (14–40 ng/mL) and in LALD (4.5–14 ng/mL) (Fig. 3B).

#### 3.4.2 Acidic pathway:- 7-OC

Considering next the pathway from 7-OC, authentic standards are available for 26H,7O-C and 3βH,7O-CA allowing their definitive identification. 26H,7O-C is present in control and carrier plasma (≤0.5 ng/mL) and is found to be elevated in NPC (1–2.5 ng/mL), NPB (1–2 ng/mL) but not LALD (<0.5 ng/mL) plasma (Fig. 5B). The 7-oxo acid, 26H,7O-CA, is present in plasma from healthy controls and carriers (1–1.5 ng/mL) but greatly elevated in plasma from NPC (13.5–60.5 ng/mL), NPB (39–60 ng/mL) and LALD (9.5–52 ng/mL) (Fig. 5C). An authentic standard is not available for 3β,24-dihydroxy-7-oxocholest-5-en-26-oic acid (3β,24-diH,7O-CA), however, based on exact mass, retention time and MS^3^ spectra it was presumptively identified in two of the three NPC (0–0.5 ng/mL), all three NPB (0.5–1.5 ng/mL) and both LALD (1–3 ng/mL) plasma samples (Fig. 5D, see Fig. 4G in Ref. [30]). 3β,24-diH,7O-CA was absent or of very low abundance in control and carrier plasma samples (<0.2 ng/mL). No authentic standard was available for 3βH,7O-Δ^5^-BA. It was presumptively identified in control and carrier (≤0.5 ng/mL), NPC (1.5–6.5 ng/mL), NPB (2–6.5 ng/mL) and LALD (1–5.5 ng/mL) plasma (Fig. 5E, see Fig. 5D in Ref. [30]).

#### *3.4.3* *Metabolite ratios: 7O/7*β

Unlike the situation for 7-OC and 7β-HC which are in a concentration ratio of about 2.5 for both NPC and NPB patents, the 7β-hydroxy acids, 3β,7β-diHCA and 3β,7β-diH-Δ^5^-BA, are more abundant than their 3-oxo analogues 3βH,7O-CA and 3βH,7O-Δ^5^-BA in the ratio of about 3.5 and 8 for the C_27_ (NPC, 3.5 ± 0.4; NPB, 3.1 ± 0.8) and C_24_ (NPC, 9.2 ± 2.7; NPB, 6.6 ± 0.2) acids, respectively. Both ratios are also greater than 1 for the LALD patients (C_27_, 1.1; C_24_, 3.43) and carriers (C_27_, 3.1 ± 0.7; C_24_, 3.5 ± 1.9) and controls. In contrast to the situation for 3β,5α,6β-triHCa, where concentrations are higher in NPB than NPC plasma (see Supplemental Table S1) the concentration ranges of the acids 3β,7β-diHCA and 3βH,7O-CA both overlap for the two disorders. In NPB plasma other acids 3β-HCA, 3β,7α-diHCA and 7αH,3O-CA were higher in NPB than in NPC plasma, as was their precursor 26-HC. An absence of a similar trend for 3β,7β-diHCA and 3βH,7O-CA suggests that their formation is not dependent on CYP27A1 activity, but rather substrate availability.

## 4 Discussion

The observation here and elsewhere of elevated levels of 3β,5α,6β-triol and 7-OC in the lysosomal storage diseases NPC and NPB points to their endogenous formation [22]. While 7-OC can be formed enzymatically from 7-DHC in SLOS [43], there is little convincing evidence for the enzymatic formation of 5,6-EC, the precursor of 3β,5α,6β-triol, hence it is likely that it is formed through free radical reactions *in vivo.* The absence of high levels of 7-DHC in NPC and NPB also points to the formation of 7-OC via *in vivo* free radical reactions.

### *4.1* *Formation of 3*β*,5α,6*β*-triHBA*

Until recently little was known about the metabolism of 5,6-EC. It is established that 5,6-EC can be enzymatically converted to the 3β,5α,6β-triol by ChEH [35], but only in 2016 was it shown that the triol could be converted to the bile acid 3β,5α,6β-triHBA in man [28,29]. A similar product has also been proposed to be generated in rat [44,45], however, a description of the pathway from 3β,5α,6β-triol to 3β,5α,6β-triHBA has not previously been reported.

There are two major pathways of bile acid biosynthesis, the neutral and acidic pathways, and two more minor pathways, the sterol 24-hydroxylase and 25-hydroxylase pathways [37]. The neutral pathway is initiated by 7α-hydroxylation of cholesterol by CYP7A1, but in the metabolism of 3β,5α,6β-triol the absence of a 7α-hydroxy group in the ultimate bile acid argues against operation of this pathway. The cholesterol 24-hydroxylase pathway is also an unlikely route for 3β,5α,6β-triol metabolism as this starts with a reaction catalysed by CYP46A1, predominantly expressed in brain [37]. This leaves the acidic and 25-hydroxylase pathways for biosynthesis of 3β,5α,6β-triHBA from 3β,5α,6β-triol. As in the present study we have identified most of the intermediates in a novel branch of the acidic pathway from 3β,5α,6β-triol to 3β,5α,6β-triHBA in plasma, it is highly likely that this is the pathway followed (Fig. 4).

### *4.2* *Formation of 3*β*H,7O-Δ*^*5*^*-BA and 3*β*,7*β*-diH-Δ*^*5*^*-BA*

The metabolism of 7-OC has been the subject of greater interest than that of 3β,5α,6β-triol [40,41,46], as the former compound is found in atherosclerotic lesions [47]. 7-OC can be metabolised to 26H,7-OC and 3βH,7O-CA [40,41], hence it likely to also follow another branch of the acidic pathway to generate 3βH,7O-Δ^5^-BA. The data reported here confirms this hypothesis as judged by the preponderance of relevant pathway intermediates identified in plasma of NPC and NPB patients. 7-OC can alternatively be converted to 7β-HC [[5], [6], [7], [8]] and this oxysterol may similarly fall into a related branch of the acidic pathway with the formation of 3β,7β-diH-Δ^5^-BA. These 7-OC and 7β-HC branches of the acidic may be interconvertible through HSD11B1 which can convert 7-oxo to 7β-hydroxy groups and HSD11B2 which can catalyse the reverse reactions (Fig. 4). In fact, in an early study Alvelius et al. identified both 3βH,7O-Δ^5^-BA and 3β,7β-diH-Δ^5^-BA as their sulphate and glycine conjugates in urine of an NPC patient [26], although the plasma pattern of oxysterols was apparently normal. In the present study, the ratio of 7-oxo- to 7β-hydroxy-sterols may provide an insight into the activity of HSD11B enzymes. HSD11B1 is the enzyme responsible for reducing 7-OC to 7β-HC in mouse and man [5,7], while HSD11B2 can also catalyse the reverse reaction [9]. The HSD11B1 enzyme also catalyses the reduction of cortisone to cortisol in man and of 11-dehydrocorticosterone to corticosterone in mouse, the reduced metabolites being ligands to the glucocorticoid receptor. On the other hand, HSD11B2 is the enzyme that oxidises cortisol to cortisone and has a similar activity towards 7β-HC and also 7β,25-dihydroxycholesterol [9,48,49]. Data from the present study indicates that as the acidic pathway proceeds the ratio of 7β-hydroxy to 7-oxo metabolites increase, this is true for patients, controls and carriers. This may be explained by the HSD11B reductase having a dominant effect over the oxidase as the pathway descends.

HSD11B2 also has activity towards 3β,5α,6β-triol, oxidising it to the oncometabolite 3β,5α-diHC-6O, while HSD11B1 catalyses the reverse reaction [11]. It appears that the enzymes ChEH, HSD11B1 and HSD11B2 and CYP27A1 sit at a fulcrum balancing the formation from 5α,6-EC of the tumour suppressor DDA and from 5,6-EC, through 3β,5α,6β-triol, the oncometabolite 3β,5α-diHC-6O or the bile acid 3β,5α,6β-triHBA. It should also be noted that 26H,7O-C activates the Hh signalling pathway, constitutive activation of which is linked to tumorigenesis further connecting the HSD11B and CYP27A1 enzymes to oncology.

In light of the low number of patient samples analysed the diagnostic value of bile acid precursors can only be speculated on. However, the high abundance of the C_27_ acids 3β,5α,6β-triHCa, 3β,7β-diHCA (Figure 3D) and 3βH,7O-CA (Fig. 5C) in NPC and NPB plasma suggests that these three acids in combination may diagnose these disorders. It may not be possible to distinguish these diseases from LALD as the three acids are elevated in this disorder also.

## 5 Conclusion

In man, most of the primary bile acids are synthesised in the liver through the neutral pathway of bile acid biosynthesis. The acidic pathway can proceed extrahepatically [37], while quantitatively less important in adult this pathway may be dominant in neonates [50]. We show here that novel branches of the acidic pathway, starting from oxysterols formed non-enzymatically, are important for the formation of unusual bile acids in patients with lysosomal storage disease. In healthy controls, the acidic pathway also proceeds to generate unusual 7β-hydroxy and 7-oxo bile acids but these are quantitatively minor.

## References

[1] Schroepfer G.J. (2000). Oxysterols: modulators of cholesterol metabolism and other processes. Physiol. Rev..

[2] Bjorkhem I. (2013). Five decades with oxysterols. Biochimie.

[3] Griffiths W.J., Crick P.J., Wang Y. (2013). Methods for oxysterol analysis: past, present and future. Biochem. Pharmacol..

[4] Shinkyo R., Xu L., Tallman K.A., Cheng Q., Porter N.A., Guengerich F.P. (2011). Conversion of 7-dehydrocholesterol to 7-ketocholesterol is catalyzed by human cytochrome P450 7A1 and occurs by direct oxidation without an epoxide intermediate. J. Biol. Chem..

[5] Hult M., Elleby B., Shafqat N., Svensson S., Rane A., Jornvall H., Abrahmsen L., Oppermann U. (2004). Human and rodent type 1 11beta-hydroxysteroid dehydrogenases are 7beta-hydroxycholesterol dehydrogenases involved in oxysterol metabolism. Cell. Mol. Life Sci..

[6] Larsson H., Bottiger Y., Iuliano L., Diczfalusy U. (2007). In vivo interconversion of 7beta-hydroxycholesterol and 7-ketocholesterol, potential surrogate markers for oxidative stress. Free Radic. Biol. Med..

[7] Mitic T., Shave S., Semjonous N., McNae I., Cobice D.F., Lavery G.G., Webster S.P., Hadoke P.W., Walker B.R., Andrew R. (2013). 11beta-Hydroxysteroid dehydrogenase type 1 contributes to the balance between 7-keto- and 7-hydroxy-oxysterols in vivo. Biochem. Pharmacol..

[8] Schweizer R.A., Zurcher M., Balazs Z., Dick B., Odermatt A. (2004). Rapid hepatic metabolism of 7-ketocholesterol by 11beta-hydroxysteroid dehydrogenase type 1: species-specific differences between the rat, human, and hamster enzyme. J. Biol. Chem..

[9] Raleigh D.R., Sever N., Choksi P.K., Sigg M.A., Hines K.M., Thompson B.M., Elnatan D., Jaishankar P., Bisignano P., Garcia-Gonzalo F.R., Krup A.L., Eberl M., Byrne E.F.X., Siebold C., Wong S.Y., Renslo A.R., Grabe M., McDonald J.G., Xu L., Beachy P.A., Reiter J.F. (2018). Cilia-associated oxysterols activate smoothened. Mol. Cell..

[10] Khalifa S.A., de Medina P., Erlandsson A., El-Seedi H.R., Silvente-Poirot S., Poirot M. (2014). The novel steroidal alkaloids dendrogenin A and B promote proliferation of adult neural stem cells. Biochem. Biophys. Res. Commun..

[11] Voisin M., de Medina P., Mallinger A., Dalenc F., Huc-Claustre E., Leignadier J., Serhan N., Soules R., Segala G., Mougel A., Noguer E., Mhamdi L., Bacquie E., Iuliano L., Zerbinati C., Lacroix-Triki M., Chaltiel L., Filleron T., Cavailles V., Al Saati T., Rochaix P., Duprez-Paumier R., Franchet C., Ligat L., Lopez F., Record M., Poirot M., Silvente-Poirot S. (2017). Identification of a tumor-promoter cholesterol metabolite in human breast cancers acting through the glucocorticoid receptor. Proc. Natl. Acad. Sci. U. S. A..

[12] Porter F.D., Scherrer D.E., Lanier M.H., Langmade S.J., Molugu V., Gale S.E., Olzeski D., Sidhu R., Dietzen D.J., Fu R., Wassif C.A., Yanjanin N.M., Marso S.P., House J., Vite C., Schaffer J.E., Ory D.S. (2010). Cholesterol oxidation products are sensitive and specific blood-based biomarkers for Niemann-Pick C1 disease. Sci. Transl. Med..

[13] Jiang X., Sidhu R., Porter F.D., Yanjanin N.M., Speak A.O., te Vruchte D.T., Platt F.M., Fujiwara H., Scherrer D.E., Zhang J., Dietzen D.J., Schaffer J.E., Ory D.S. (2011). A sensitive and specific LC-MS/MS method for rapid diagnosis of Niemann-Pick C1 disease from human plasma. J. Lipid Res..

[14] Lin N., Zhang H., Qiu W., Ye J., Han L., Wang Y., Gu X. (2014). Determination of 7-ketocholesterol in plasma by LC-MS for rapid diagnosis of acid SMase-deficient Niemann-Pick disease. J. Lipid Res..

[15] Pajares S., Arias A., Garcia-Villoria J., Macias-Vidal J., Ros E., de las Heras J., Giros M., Coll M.J., Ribes A. (2015). Cholestane-3beta,5alpha,6beta-triol: high levels in Niemann-Pick type C, cerebrotendinous xanthomatosis, and lysosomal acid lipase deficiency. J. Lipid Res..

[16] Klinke G., Rohrbach M., Giugliani R., Burda P., Baumgartner M.R., Tran C., Gautschi M., Mathis D., Hersberger M. (2015). LC-MS/MS based assay and reference intervals in children and adolescents for oxysterols elevated in Niemann-Pick diseases. Clin. Biochem..

[17] Boenzi S., Deodato F., Taurisano R., Goffredo B.M., Rizzo C., Dionisi-Vici C. (2016). Evaluation of plasma cholestane-3beta,5alpha,6beta-triol and 7-ketocholesterol in inherited disorders related to cholesterol metabolism. J. Lipid Res..

[18] Romanello M., Zampieri S., Bortolotti N., Deroma L., Sechi A., Fiumara A., Parini R., Borroni B., Brancati F., Bruni A., Russo C.V., Bordugo A., Bembi B., Dardis A. (2016). Comprehensive evaluation of plasma 7-ketocholesterol and cholestan-3beta,5alpha,6beta-triol in an Italian cohort of patients affected by niemann-pick disease due to NPC1 and SMPD1 mutations. Clin. Chim. Acta.

[19] Myers B.R., Sever N., Chong Y.C., Kim J., Belani J.D., Rychnovsky S., Bazan J.F., Beachy P.A. (2013). Hedgehog pathway modulation by multiple lipid binding sites on the smoothened effector of signal response. Dev. Cell.

[20] Soroosh P., Wu J., Xue X., Song J., Sutton S.W., Sablad M., Yu J., Nelen M.I., Liu X., Castro G., Luna R., Crawford S., Banie H., Dandridge R.A., Deng X., Bittner A., Kuei C., Tootoonchi M., Rozenkrants N., Herman K., Gao J., Yang X.V., Sachen K., Ngo K., Fung-Leung W.P., Nguyen S., de Leon-Tabaldo A., Blevitt J., Zhang Y., Cummings M.D., Rao T., Mani N.S., Liu C., McKinnon M., Milla M.E., Fourie A.M., Sun S. (2014). Oxysterols are agonist ligands of RORgammat and drive Th17 cell differentiation. Proc. Natl. Acad. Sci. U. S. A..

[21] de Medina P., Paillasse M.R., Segala G., Voisin M., Mhamdi L., Dalenc F., Lacroix-Triki M., Filleron T., Pont F., Saati T.A., Morisseau C., Hammock B.D., Silvente-Poirot S., Poirot M. (2013). Dendrogenin A arises from cholesterol and histamine metabolism and shows cell differentiation and anti-tumour properties. Nat. Commun..

[22] Wang Y., Griffiths W.J. (2018). Unravelling new pathways of sterol metabolism: lessons learned from in-born errors and cancer. Curr. Opin. Clin. Nutr. Metab. Care.

[23] Wang Y., Griffiths W.J. (2017). Oxysterol Lipidomics in Mouse and Man, Keystone Symposium, Lipidomics and Bioactive Lipids in Metabolism and Disease, Granlibakken Tahoe, Tahoe City, California, USA.

[24] Griffiths W.J., Abdel-Khalik J., Crick P.T., Ogundare M., Bigger B.W., Morris A.A., Shackleton C.H., Clayton P.T., Sjovall J., Bjorkhem I., Wang Y. (2016). Bile Acid Biosynthesis Avoiding Cholesterol, XXIV International Bile Acid Meeting: Bile Acids in Health and Disease, Düsseldorf, Germany.

[25] Griffiths W.J., Abdel-Khalik J., Crick P.T., Wang Y. (2016). Unravelling new pathways of sterol metabolism, LE STUDIUM Conference, Lipids, nanotechnology and cancer., Hôtel de Ville de Tours, France.

[26] Alvelius G., Hjalmarson O., Griffiths W.J., Bjorkhem I., Sjovall J. (2001). Identification of unusual 7-oxygenated bile acid sulfates in a patient with Niemann-Pick disease, type C. J. Lipid Res..

[27] Zielinski Z.A., Pratt D.A. (2016). Cholesterol autoxidation revisited: debunking the dogma associated with the most vilified of lipids. J. Am. Chem. Soc..

[28] Mazzacuva F., Mills P., Mills K., Camuzeaux S., Gissen P., Nicoli E.R., Wassif C., Te Vruchte D., Porter F.D., Maekawa M., Mano N., Iida T., Platt F., Clayton P.T. (2016). Identification of novel bile acids as biomarkers for the early diagnosis of Niemann-Pick C disease. FEBS Lett..

[29] Jiang X., Sidhu R., Mydock-McGrane L., Hsu F.F., Covey D.F., Scherrer D.E., Earley B., Gale S.E., Farhat N.Y., Porter F.D., Dietzen D.J., Orsini J.J., Berry-Kravis E., Zhang X., Reunert J., Marquardt T., Runz H., Giugliani R., Schaffer J.E., Ory D.S. (2016). Development of a bile acid-based newborn screen for Niemann-Pick disease type C. Sci. Transl. Med..

[30] Griffiths W.J., Gilmore I., Yutuc E., Abdel-Khalik J., Crick P.J., Hearn T., Dickson A., Bigger B.W., Wu T.H., Goenka A., Ghosh A., Jones S.A., Wang Y. (2018). Identification of unusual oxysterols and bile acids with 7-oxo or 3beta,5alpha,6beta-trihydroxy functions in human plasma by charge-tagging mass spectrometry with multistage fragmentation. J. Lipid Res..

[31] Crick P.J., William Bentley T., Abdel-Khalik J., Matthews I., Clayton P.T., Morris A.A., Bigger B.W., Zerbinati C., Tritapepe L., Iuliano L., Wang Y., Griffiths W.J. (2015). Quantitative charge-tags for sterol and oxysterol analysis. Clin. Chem..

[32] Crick P.J., Bentley T.W., Wang Y., Griffiths W.J. (2015). Revised sample preparation for the analysis of oxysterols by enzyme-assisted derivatisation for sterol analysis (EADSA). Anal. Bioanal. Chem..

[33] Wang Y., Sousa K.M., Bodin K., Theofilopoulos S., Sacchetti P., Hornshaw M., Woffendin G., Karu K., Sjovall J., Arenas E., Griffiths W.J. (2009). Targeted lipidomic analysis of oxysterols in the embryonic central nervous system. Mol. Biosyst..

[34] Murphy R.C., Johnson K.M. (2008). Cholesterol, reactive oxygen species, and the formation of biologically active mediators. J. Biol. Chem..

[35] Silvente-Poirot S., Poirot M. (2012). Cholesterol epoxide hydrolase and cancer. Curr. Opin. Pharmacol..

[36] Quehenberger O., Armando A.M., Brown A.H., Milne S.B., Myers D.S., Merrill A.H., Bandyopadhyay S., Jones K.N., Kelly S., Shaner R.L., Sullards C.M., Wang E., Murphy R.C., Barkley R.M., Leiker T.J., Raetz C.R., Guan Z., Laird G.M., Six D.A., Russell D.W., McDonald J.G., Subramaniam S., Fahy E., Dennis E.A. (2010). Lipidomics reveals a remarkable diversity of lipids in human plasma. J. Lipid Res..

[37] Russell D.W. (2003). The enzymes, regulation, and genetics of bile acid synthesis. Annu. Rev. Biochem..

[38] Hunt M.C., Solaas K., Kase B.F., Alexson S.E. (2002). Characterization of an acyl-coA thioesterase that functions as a major regulator of peroxisomal lipid metabolism. J. Biol. Chem..

[39] Hunt M.C., Yamada J., Maltais L.J., Wright M.W., Podesta E.J., Alexson S.E. (2005). A revised nomenclature for mammalian acyl-CoA thioesterases/hydrolases. J. Lipid Res..

[40] Lyons M.A., Brown A.J. (2001). Metabolism of an oxysterol, 7-ketocholesterol, by sterol 27-hydroxylase in HepG2 cells. Lipids.

[41] Heo G.Y., Bederman I., Mast N., Liao W.L., Turko I.V., Pikuleva I.A. (2011). Conversion of 7-ketocholesterol to oxysterol metabolites by recombinant CYP27A1 and retinal pigment epithelial cells. J. Lipid Res..

[42] Griffiths W.J., Hearn T., Crick P.J., Abdel-Khalik J., Dickson A., Yutuc E., Wang Y. (2017). Charge-tagging liquid chromatography-mass spectrometry methodology targeting oxysterol diastereoisomers. Chem. Phys. Lipids.

[43] Bjorkhem I., Diczfalusy U., Lovgren-Sandblom A., Starck L., Jonsson M., Tallman K., Schirmer H., Ousager L.B., Crick P.J., Wang Y., Griffiths W.J., Guengerich F.P. (2014). On the formation of 7-ketocholesterol from 7-dehydrocholesterol in patients with CTX and SLO. J. Lipid Res..

[44] Kikuchi S., Imai Y., Suzuoki Z., Matsu T., Noguchi S. (1968). Biologic studies of cholestane-3-beta,5-alpha,6-beta-triol and its derivatives. 3. The metabolic fate and metabolites of cholestane-3-beta,5-alpha,6-beta-triol in animals. J. Pharmacol. Exp. Ther..

[45] Roscoe H.G., Goldstein R., Fahrenbach M.J. (1968). Metabolism of cholestane-3-beta,5-alpha,6-beta-triol. I. The fate of cholestanetriol in the rat. Biochem. Pharmacol..

[46] Brown A.J., Watts G.F., Burnett J.R., Dean R.T., Jessup W. (2000). Sterol 27-hydroxylase acts on 7-ketocholesterol in human atherosclerotic lesions and macrophages in culture. J. Biol. Chem..

[47] Brown A.J., Jessup W. (1999). Oxysterols and atherosclerosis. Atherosclerosis.

[48] Stewart P.M., Krozowski Z.S. (1999). 11 beta-Hydroxysteroid dehydrogenase. Vitam. Horm..

[49] Beck K.R., Kanagaratnam S., Kratschmar D.V., Birk J., Yamaguchi H., Sailer A.W., Seuwen K., Odermatt A. (2019). Enzymatic interconversion of the oxysterols 7beta,25-dihydroxycholesterol and 7-keto,25-hydroxycholesterol by 11beta-hydroxysteroid dehydrogenase type 1 and 2. J. Steroid Biochem. Mol. Biol..

[50] Setchell K.D., Schwarz M., O'Connell N.C., Lund E.G., Davis D.L., Lathe R., Thompson H.R., Weslie Tyson R., Sokol R.J., Russell D.W. (1998). Identification of a new inborn error in bile acid synthesis: mutation of the oxysterol 7alpha-hydroxylase gene causes severe neonatal liver disease. J. Clin. Investig..

